# Supplementary material for: Hereditary Hyperferritinemia Cataract Syndrome: Ferritin L Gene and Physiopathology behind the Disease—Report of New Cases
Source: Int J Mol Sci. 2021 May 21;22(11):5451. doi: 10.3390/ijms22115451 (PMC8196845; doi:10.3390/ijms22115451)
Supplement: Supplementary file 1 [file ijms-22-05451-s001.zip › ijms-1219121-supplementary.pdf]

Supplementary Material

Hereditary Hyperferritinemia Cataract Syndrome: Ferritin L Gene and Physiopathology behind the Disease—Report of New Cases

Table S1. Reported mutations in HHCS disease.

N	HGVS Nomenclature	Mutation Type	Mutation Position	First Publication	Old Nomenclature
1	c.-216C>A	IRE regulatory	Promoter *FTL*	[1]	NA
2	c.-193C>G + c.-160A>G	IRE regulatory	lower stem + hexanucleotide loop	[2]	+7C>G & +40A>G
3	c.-190C>T	IRE regulatory	lower stem	[3]	+10C>U
4	c.-186C>G	IRE regulatory	lower stem	[4]	+14C>G
5	c.-184C>T	IRE regulatory	lower stem	[3]	+16C>U
6	c.-182C>T + c.-178T>G	IRE regulatory	lower stem	[5]	Paiva-2 + 18C>U & 22U>G
7	c.-176T>C	IRE regulatory	lower stem	[6]	+24U>C
8	c.-171C>G	IRE regulatory	lower stem	[7]	Torino +29C>G
9	c.-168G>A	IRE regulatory	lower stem	[5]	Pavia-1 +32G>A
10	c.-168G>C	IRE regulatory	lower stem	[8]	Baltimore-1 +32G>C
11	c.-168G>T	IRE regulatory	lower stem	[9]	Paris-2 or Milano-1 +32G>U
12	c.-167C>A	IRE regulatory	C bulge	[10]	Paris +33C>A
13	c.-167C>T	IRE regulatory	C bulge	[11]	Madrid or Philadelphia +33C>U
14	c.-166T>C	IRE regulatory	upper stem	[12]	Paris +34U>C
15	c.-164C>A	IRE regulatory	upper stem	[13]	London-2 +36C>A
16	c.-164C>G	IRE regulatory	upper stem	[3]	Milano +36C>G
17	c.-164C>T	IRE regulatory	upper stem	[14]	Badalona +36C>U
18	c.-163A>C	IRE regulatory	upper stem	[15]	Pavia +37A>C
19	c.-163A>G	IRE regulatory	upper stem	[3]	Milano +37A>G
20	c.-163A>T	IRE regulatory	upper stem	[16]	Zaragoza +37A>U
21	c.-161C>A	IRE regulatory	hexanucleotide loop	[17]	Geelong +39C>A
22	c.-161C>G	IRE regulatory	hexanucleotide loop	[18]	Paris +39C>G
23	c.-161C>T	IRE regulatory	hexanucleotide loop	[13]	London-1 +39C>U
24	c.-160A>G	IRE regulatory	hexanucleotide loop	[19]	Paris-1 or Montpellier-1 +40A>G
25	c.-160A>G + c.-159G>C	IRE regulatory	hexanucleotide loop	[4]	Paris-1 or Montpellier-1 +40A>G & Verona-1 +41G>C
26	c.-159G>C	IRE regulatory	hexanucleotide loop	[20]	Verona-1 +41G>C
27	c.-157G>A	IRE regulatory	hexanucleotide loop	[21]	Salt Lake City +43G>A
28	c.-154T>G	IRE regulatory	upper stem	[22]	+46U>G
29	c.-153G>A	IRE regulatory	upper stem	[12]	Paris +47G>A
30	c.-151A>C	IRE regulatory	lower stem	[2]	+49A>C
31	c.-151A>G	IRE regulatory	lower stem	[23]	Ghent +49A > G
32	c.-150C>A	IRE regulatory	lower stem	[24]	+50C>A
33	c.-149G>C	IRE regulatory	lower stem	[25]	Torino +51G>C
34	c.-148G>C	IRE regulatory	lower stem	[14]	Heidelberg +52G>C
35	c.-144A>T	IRE regulatory	lower stem	[15]	Paris +56A>U
36	c.-110C>T	IRE regulatory	5′ UTR	[3]	+90C>U
37	c.-220_-196del25	IRE regulatory	new transcription starting site (resulting IRE lacks nt 1-24)	[26]	NA
38	c.-190-162del29	IRE regulatory	eliminating IRE	[27]	Verona-2 +10_38del29
39	c.-182_-174 delCGGGTCTGT insAGGGGCCGG $	IRE regulatory	eliminating part of lower stem	[28]	+18_+26 delCGGGTCTGT insAGGGGCCGG
40	c.-178_-173del6	IRE regulatory	eliminating part of lower stem	[29]	+22_27del6
41	c.-168_-165delGCTT	IRE regulatory	eliminating C bulge	[30]	+32_35delGCTT
42	c.-164_158del7	IRE regulatory	eliminating part of hexanucleotide loop	[31]	Esplugues +36_42d
43	c.-161delC	IRE regulatory	eliminating IRE	[32]	+39delC
44	c.-162_-161delCA	IRE regulatory	eliminating part of hexanucleotide loop	[33]	+38_39del AC
45	c.-158_-143del16	IRE regulatory	eliminating part of hexanucleotide loop	[33]	+42_57del16

46	c.-153_-152 delGGinsCT	IRE regulatory	eliminating part of upper stem	[34]	+47_48delGGinsCT
47	c.-44delT	IRE regulatory	eliminating IRE	[3]	+176delT

References

1. Faniello, M.C.; Di Sanzo, M.; Quaresima, B.; Nisticò, A.; Fregola, A.; Grosso, M.; Cuda, G.; Costanzo, F. Bilateral cataract in a subject carrying a C to A transition in the L ferritin promoter region. *Clin. Biochem.* **2009**, *42*, 911–914.
2. Castiglioni, E.; Soriani, N.; Girelli, D.; Camaschella, C.; Spiga, I.; Della Porta, M.G.; Ferrari, M.; Cremonesi, L. High resolution melting for the identification of mutations in the iron responsive element of the ferritin light chain gene. *Clin. Chem. Lab. Med.* **2010**, *48*, 1415–1418.
3. Cremonesi, L.; Paroni, R.; Foglieni, B.; Galbiati, S.; Fermo, I.; Soriani, N.; Belloli, S.; Ruggeri, G.; Biasiotto, G.; Cazzola, M.; et al. Scanning mutations of the 5′UTR regulatory sequence of L-ferritin by denaturing high-performance liquid chromatography: Identification of new mutations. *Br. J. Haematol.* **2003**, *121*, 173–179.
4. Cremonesi, L.; Fumagalli, A.; Soriani, N.; Ferrari, M.; Levi, S.; Belloli, S.; Ruggeri, G.; Arosio, P. Double-gradient denaturing gradient gel electrophoresis assay for identification of L-ferritin iron-responsive element mutations responsible for hereditary hyperferritinemia-cataract syndrome: Identification of the new mutation C14G. *Clin. Chem.* **2001**, *47*, 491–497.
5. Cazzola, M.; Bergamaschi, G.; Tonon, L.; Arbustini, E.; Grasso, M.; Vercesi, E.; Barosi, G.; Bianchi, P.E.; Cairo, G.; Arosio, P. Hereditary hyperferritinemia-cataract syndrome: Relationship between phenotypes and specific mutations in the iron-responsive element of ferritin light-chain mRNA. *Blood* **1997**, *90*, 814–821.
6. Rüfer, A.; Howell, J.P.; Lange, A.P.; Yamamoto, R.; Heuscher, J.; Gregor, M.; Wuillemin, W.A. Hereditary hyperferritinemia-cataract syndrome (HHCS) presenting with iron deficiency anemia associated with a new mutation in the iron responsive element of the L ferritin gene in a Swiss family. *Eur. J. Haematol.* **2011**, *87*, 274–278.
7. Bosio, S.; Campanella, A.; Gramaglia, E.; Porporato, P.E.; Longo, F.; Cremonesi, L.; Levi, S.; Camaschella, C. C29G in the iron-responsive element of L-ferritin: A new mutation associated with hyperferritinemia-cataract. *Blood Cells Mol. Dis.* **2004**, *33*, 31–34.
8. Kato, G.J.; Casella, F. L-ferritin-Baltmore-1: A novel mutation in the iron response element (C32G) as a cause of the hyperferritinemia-cataract syndrome. *Blood* **1999**, 94, 407a.
9. Martin, M.E.; Fargion, S.; Brissot, P.; Pellat, B.; Beaumont, C. A point mutation in the bulge of the iron-responsive element of the L ferritin gene in two families with the hereditary hyperferritinemia-cataract syndrome. *Blood* **1998**, *91*, 319–323.
10. Durupt, S.; Durieu, I.; Salles, B.; Beaumont, C.; Hot, A.; Rousset, H.; Vital Durand, D. A potential etiology of elevated ferritin: Hyperferritinemia-cataract syndrome. *Rev. Med. Interne* **2001**, *22*, 83–85.
11. Balas, A.; Aviles, M.J.; Garcia-Sanchez, F.; Vicario, J.L. Description of a new mutation in the L-ferrin iron-responsive element associated with hereditary hyperferritinemia-cataract syndrome in a Spanish family. *Blood* **1999**, *93*, 4020–4021.
12. Hetet, G.; Devaux, I.; Soufir, N.; Grandchamp, B.; Beaumont, C. Molecular analyses of patients with hyperferritinemia and normal serum iron values reveal both L ferritin IRE and 3 new ferroportin (slc11A3) mutations. *Blood* **2003**, *102*, 1904–1910.
13. Mumford, A.D.; Vulliamy, T.; Lindsay, J.; Watson, A. Hereditary hyperferritinemia-cataract syndrome: Two novel mutations in the L-ferritin iron-responsive element. *Blood* **1998**, *91*, 367–368.
14. Luscieti, S.; Tolle, G.; Aranda, J.; Campos, C.B.; Risse, F.; Morán, É.; Muckenthaler, M.U.; Sánchez, M. Novel mutations in the ferritin-L iron-responsive element that only mildly impair IRP binding cause hereditary hyperferritinaemia cataract syndrome. *Orphanet J. Rare Dis.* **2013**, *8*, 30.
15. Ferrari, F.; Foglieni, B.; Arosio, P.; Camaschella, C.; Daraio, F.; Levi, S.; Garcia Erce, J.A.; Beaumont, C.; Cazzola, M.; Ferrari, M.; et al. Microelectronic DNA chip for hereditary hyperferritinemia cataract syndrome, a model for large-scale analysis of disorders of iron metabolism. *Hum. Mutat*. **2006**, *27*, 201–208.
16. García Erce, J.A.; Cortés, T.; Cremonesi, L.; Cazzola, M.; Pérez-Lungmus, G.; Giralt, M. Hyperferritinemia-cataract syndrome associated to the HFE gene mutation. Two new Spanish families and a new mutation (A37T: "Zaragoza"). *Med. Clin.* **2006**, *127*, 55–58.
17. McLeod, J.L.; Craig, J.; Gumley, S.; Roberts, S.; Kirkland, M.A. Mutation spectrum in Australian pedigrees with hereditary hyperferritinaemia-cataract syndrome reveals novel and de novo mutations. *Br. J. Haematol.* **2002**, *118*, 1179–1182.
18. Garderet, L.; Hermelin, B.; Gorin, N.C.; Rosmorduc, O. Hereditary hyperferritinemia-cataract syndrome: A novel mutation in the iron-responsive element of the L-ferritin gene in a French family. *Am. J. Med.* **2004**, *117*, 138–139.
19. Beaumont, C.; Leneuve, P.; Devaux, I.; Scoazec, J.-Y.; Berthier, M.; Loiseau, M.-N.; Grandchamp, B.; Bonneau, D. Mutation in the iron responsive element of the L ferritin mRNA in a family with dominant hyperferritinaemia and cataract. *Nat. Genet.* **1995**, *11*, 444–446.
20. Meneses, F.G.A.; Schnabel, B.; Silva, I.D.C.G.; Alberto, F.L.; Toma, L.; Nader, H.B.; Lopes, C.C. Identification of the mutations associated with hereditary hyperferritinemia cataract syndrome and hemochromatosis in a Brazilian family. *Clin. Genet.* **2011**, *79*, 189–192.
21. Phillips, J.D.; Warby, C.A.; Kushner, J.P. Identification of a novel mutation in the L-ferritin IRE leading to hereditary hyperferritinemia-cataract syndrome. *Am. J. Med. Genet. A* **2005**, *134A*, 77–79.
22. Messa, E.; Pellegrino, R.; Palmieri, A.; Carturan, S.; Cilloni, D.; Saglio, G.; Roetto, A. Identification of a novel mutation in the L ferritin iron-responsive element causing hereditary hyperferritinemia-cataract syndrome. *Acta Haematol*. **2009**, *122*, 223–225.

23. Van de Sompele, S.; Pécheux, L.; Couso, J.; Meunier, A.; Sanchez, M.; De Baere, E. Functional characterization of a novel non-coding mutation "Ghent +49A > G" in the iron-responsive element of L-ferritin causing hereditary hyperferritinaemia-cataract syndrome. *Sci. Rep.* **2017**, *7*, 18025.
24. Gonzalez-Huerta, L.; Ramirez-Sanchez, V.; Rivera-Vega, M.; Messina-Baas, O.; Cuevas-Covarrubias, S. A family with hereditary hyperferritinaemia cataract syndrome: Evidence of incomplete penetrance and clinical heterogeneity. *Br. J. Haematol.* **2008**, *143*, 596–598.
25. Camaschella, C.; Zecchina, G.; Lockitch, G.; Roetto, A.; Campanella, A.; Arosio, P.; Levi, S. A new mutation (G51C) in the iron-responsive element (IRE) of L-ferritin associated with hyperferritinaemia-cataract syndrome decreases the binding affinity of the mutated IRE for iron-regulatory proteins. *Br. J. Haematol.* **2000**, *108*, 480–482.
26. Burdon, K.P.; Sharma, S.; Chen, C.S.; Dimasi, D.P.; Mackey, D.A.; Craig, J.E. A novel deletion in the FTL gene causes hereditary hyperferritinemia cataract syndrome (HHCS) by alteration of the transcription start site. *Hum. Mutat.* **2007**, *28*, 742.
27. Girelli, D.; Corrocher, R.; Bisceglia, L.; Olivieri, O.; Zelante, L.; Panozzo, G.; Gasparini, P. Hereditary hyperferritinemia-cataract syndrome caused by a 29-base pair deletion in the iron responsive element of ferritin L.-subunit gene. *Blood* **1997**, *90*, 2084–2088.
28. (Lenzhofer, M.; Schroedl, F.; Trost, A.; Kaser-Eichberger, A.; Wiedemann, H.; Strohmaier, C.; Hohensinn, M.; Strasser, M.; Muckenthaler, M.U.; Grabner, G.; et al. Aqueous humor ferritin in hereditary hyperferritinemia cataract syndrome. *Optom. Vis. Sci.* **2015**, *92* (Suppl. 1), S40–S47.
29. Cazzola, M.; Foglieni, B.; Bergamaschi, G.; Levi, S.; Lazzarino, M.; Arosio, P. A novel deletion of the L-ferritin iron-responsive element responsible for severe hereditary hyperferritinaemia-cataract syndrome. *Br. J. Haematol.* **2002**, *116*, 667–670.
30. Garber, I.; Pudek, M. A novel deletion in the iron-response element of the L-ferritin gene, causing hyperferritinaemia cataract syndrome. *Ann. Clin. Biochem.* **2014**, *51*, 710–713.
31. Cadenas, B.; Fita-Torró, J.; Bermúdez-Cortés, M.; Hernandez-Rodriguez, I.; Fuster, J.L.; Llinares, M.E.; Galera, A.M.; Romero, J.L.; Pérez-Montero, S.; Tornador, C.; et al. L-Ferritin: One Gene, Five Diseases; from Hereditary Hyperferritinemia to Hypoferritinemia-Report of New Cases. *Pharmaceuticals* **2019**, *12*, 17.
32. Muñoz-Muñoz, J.; Cuadrado-Grande, N.; Moreno-Carralero, M.I.; Hoyos-Sanabria, B.; Manubes-Guarch, A.; González, A.F.; Tejada-Palacios, P.; Del-Castillo-Rueda, A.; Moran-Jimenez, M.J. Hereditary hyperferritinemia cataract syndrome in four patients with mutations in the IRE of the FTL gene. *Clin. Genet.* **2013**, *83*, 491–493.
33. Giansily, M.; Beaumont, C.; Desveaux, C.; Hetet, G.; Schved, J.-F.; Aguilar-Martinez, P. Denaturing gradient gel electrophoresis screening for mutations in the hereditary hyperferritinaemia cataract syndrome. *Br. J. Haematol.* **2001**, *112*, 51–54.
34. Mattila, R.; Sainio, A.; Järveläinen, M.; Pursiheimo, J.; Järveläinen, H. A novel double nucleotide variant in the ferritin-L iron-responsive element in a Finnish patient with hereditary hyperferritinaemia-cataract syndrome. *Acta Ophthalmol.* **2018**, *96*, 95–99.
